# “A manager in the minds of doctors:” a comparison of new modes of control in European hospitals

**DOI:** 10.1186/1472-6963-13-246

**Published:** 2013-07-02

**Authors:** Ellen Kuhlmann, Viola Burau, Tiago Correia, Roman Lewandowski, Christos Lionis, Mirko Noordegraaf, Jose Repullo

**Affiliations:** 1 Institute of Economics, Labour and Culture, Goethe-University Frankfurt, Frankfurt, Germany; 2 Department of Political Science, University of Aarhus, Aarhus, Denmark; 3 Department of Sociology, University Institute of Lisbon, Lisbon, Portugal; 4 Voivodeship Rehabilitation Hospital for Children in Ameryka, Poland; 5 Clinic of Social and Family Medicine, University of Crete, Greece, School of Medicine, Crete, Greece; 6 Utrecht School of Governance, Utrecht, The Netherlands; 7 Institute for Health, Carlos III, Madrid, Spain

**Keywords:** European Comparison, Hospital Governance, Doctors In Management, Professionalism, Cost And Quality Management, Coordination Of Control Modes

## Abstract

**Background:**

Hospital governance increasingly combines management and professional self-governance. This article maps the new emergent modes of control in a comparative perspective and aims to better understand the relationship between medicine and management as hybrid and context-dependent. Theoretically, we critically review approaches into the managerialism-professionalism relationship; methodologically, we expand cross-country comparison towards the meso-level of organisations; and empirically, the focus is on processes and actors in a range of European hospitals.

**Methods:**

The research is explorative and was carried out as part of the FP7 COST action IS0903 Medicine and Management, Working Group 2. Comprising seven European countries, the focus is on doctors and public hospitals. We use a comparative case study design that primarily draws on expert information and document analysis as well as other secondary sources.

**Results:**

The findings reveal that managerial control is not simply an external force but increasingly integrated in medical professionalism. These processes of change are relevant in all countries but shaped by organisational settings, and therefore create different patterns of control: (1) ‘integrated’ control with high levels of coordination and coherent patterns for cost and quality controls; (2) ‘partly integrated’ control with diversity of coordination on hospital and department level and between cost and quality controls; and (3) ‘fragmented’ control with limited coordination and gaps between quality control more strongly dominated by medicine, and cost control by management.

**Conclusions:**

Our comparison highlights how organisations matter and brings the crucial relevance of ‘coordination’ of medicine and management across the levels (hospital/department) and the substance (cost/quality-safety) of control into perspective. Consequently, coordination may serve as a taxonomy of emergent modes of control, thus bringing new directions for cost-efficient and quality-effective hospital governance into perspective.

## Background

Hospital governance in European countries increasingly builds on mixed forms of governing and combines management and professional self-governance, thereby creating new more ‘hybrid’ controls that are expected to improve cost-efficiency and quality of services [1]. Currently, much of the research is concerned with the instruments of governance, often assuming linear relationships between a new policy, its implementation and the effects in practice. But overall, new policies aiming at medical management do not deliver the desired results when implemented in practice. Conry et al. [2] therefore conclude from their systematic review of hospital interventions that more theory driven interventions are needed in order to improve effective implementation. Here, our comparative study comes in and may help us to explore the determinants of new emergent forms of control.

In our study, we draw on a broad definition of ‘control’ (or ‘clinical management’ more generally) that includes a wide range of bureaucratic measurements and managerial tools, such as target setting and performance indicators, and also interventions where doctors have oversight, such as evidence-based guidelines and continuing professional development (CPD). The new emergent modes of control on the level of organisations are mapped in a cross-country comparative perspective, focusing on doctors as the target group of hospital governance and on costs and quality as the key areas of managing medical performance.

The linkage between macro-level (countries) and meso-level (organisations) governance through the lens of country comparison is innovative for two reasons: firstly, this makes it possible to move beyond a discourse of ‘hybridisation’ of medicine and management and to analyse *how* the context matters and secondly, how this creates specific forms of control. This approach may contribute to a ‘deepening’ of our understanding of complex interventions into clinical management [3] and explore new directions for cost-efficient and quality-effective hospital governance.

The concept of governance (as defined in the health policy debate and governance literature) [4-8] may serve as a helpful framework in which to re-locate theories and research from the realms of the sociology of professions and organisations as well as management studies. Placed in a broader policy context, we revise the managerialism-professionalism relationships and introduce a more flexible and integrative approach. Empirically, we set the focus on processes and actors and contribute new material on how to improve operational governance in hospitals [7] and control modes [9] in a range of European countries. Methodologically, we bring cross-country comparison to the meso-level of the organisation in a range of European countries. This brings a novel perspective into comparative research, because comparison traditionally focuses on institutions on the macro-level and often uses Anglo-American countries and/or National Health Service (NHS) systems as examples [10].

### The empirical background: opening the box of control in hospitals

Over recent years, new managerial regimes were introduced that subsequently provoked scholarly debate about the outcomes, specifically in relation to the role of doctors and professionalism, and whether the changes may bring about risks or benefits for healthcare services and for patients [11-17]. Yet, the evidence is scattered and controversial when it comes to the processes and organisational settings that create new modes of controls.

For example, a study carried out in the United States compares the leadership potential of doctors and managers using the quality of services provided in different hospitals as an indicator [18]. This study clearly confirms that doctors take over management positions, but the evidence related to the leadership potential is far from clear. An increase in managerial governance is also confirmed by Reich [19] who draws on electronic patient records and qualitative research from the US. According to this author, the new patient records are a ‘disciplinary technology’ that creates ‘disciplined doctors’ [19], p. 1021. In a similar vein, Dixon-Woods et al. [20] discuss new managerial governance tools as a threat to doctors and a kind of negative sanction to be applied if doctors are not able and willing to establish more effective self-governing control, as highlighted by the scandals in the National Health Service (NHS) in England.

Studies carried out in a range of European countries reveal variation in the transformations of control, thus drawing a more complex picture. To begin with, in the German medical profession, managerial and professional self-governing procedures are increasingly combined [21]. These developments nurture the ‘rise of a new professionalism’ that may be more inclusive [21], p. 221, Table 10.11 but it also may embody opportunities for doctors to transform and strategically use managerial tools, like evidence-based clinical guidelines and quality reports. Some specialties are better equipped than others to utilise and integrate the new clinical management in daily professional practices. Consequently, the governance changes may also impact in the occupational structure and rearrange the order and status of professional groups [21].

In a similar vein, but arguing from a Spanish perspective, Sacristán et al. [22] highlight new connections between medicine and management that, in turn, provide new chances for doctors to utilise management. As the authors put it: ‘The evolution of the discipline and the trend towards a tailored therapy suggest that health economics is not the end of clinical freedom but the start of it’ [22], p. 1; see also [23]. Tousijn [24], p. 529. in an Italian case study, also found that doctors ‘create their own managerial procedures’ rather ‘adapting’ or ‘modifying’ existing managerial procedures or ‘circumventing them’.

Drawing on material from Norway, Martinussen and Magnussen [17] add further evidence of variety of transformations underway in healthcare. The authors conclude: ‘Our findings support the view that, rather than managerialist values colonising the medical profession through a process of hybridisation, there is heterogeneity within the profession: some physician managers are adopting management values and tools, whereas others remain alienated from them’ [17,25,26]. A similar conclusion was drawn from a literature research of entrepreneurship in western hospitals. The authors identify ‘various responses’, including among others, a ‘transformative attitude towards traditional medical professionalism’ that supports entrepreneurial elements of healthcare [27,28].

A European study confirms a general trend towards new connections between management and professional self-governance that was described in single-country research or secondary analysis and adds further empirical evidence [9]. This study highlights that, ‘medicine and management are “twin forces”, and as such indicative for the new emergent controls’ [9], p. 722. What matters are the balance and the ‘specific composition of the toolset of controls’ [9], p. 723. Here, it seems to be important to take the different levels of governance into account, because ‘publicly operated hospitals increasingly have a meso-level governance structure that resembles that of a private company’ and important decisions are increasingly made on this level [7], p. 5.

In summary, existing research highlights important changes in the modes of control, but no uniform pattern of professionalism-managerialism relationships could be identified. Instead, there is growing evidence of variety, diversity and heterogeneity of the ways management and professionalism are connected, including the design of clinical management by doctors. This raises the questions what actually matters in creating new modes of control, and what are the determinants that may foster more efficient and integrated forms. Against the backdrop of a lack of any clear empirical evidence of favourable conditions and the call for more theory-led intervention strategy [2], the next section critically discusses how the scholarly theories may support our research questions.

### The theoretical background: the managerialism-professionalism relationship revisited

The US sociologist Eliot Freidson [29] is perhaps the most prominent author who theorised the relationship between doctors/professionalism and management/bureaucratic regulations from the perspective of the sociology of professions. Freidson has argued that professionalism acts as a ‘third logic’ next to rational-legal bureaucracy developed by Max Weber, which represents managerialism, and ‘Adam’s model of the free market which represents consumerism’ [29], p. 179.

The idea of a third logic is closely linked with the assumption of ‘countervailing powers’ and ‘conflict’ between professionalism and managerialism [30,31]. Although these approaches were most influential in the 1990s, they are still stressed especially in Anglo-American research. For instance, in their recent study of the medical profession in England, Dixon-Woods et al. argue: ‘The new rebalancing of the “countervailing powers” has dislodged the profession as the senior partner in the regulation of doctors, but may introduce new risk’ [20], p. 1452.

The countervailing powers approach embodies the problems of dichotomy that cannot adequately grasp more inclusive and mixed emergent models and that underestimates the transformative capacities of professionalism [8,28]. Johnson [32] was among the first who proposed to overcome the static and contradictory conception of external regulation and professionalism by taking up the Foucaudian concept of governmentality. This approach opens up new perspectives directing our attention toward social contexts and transformativity of professionalism. It also has paved the way for conceptualising medical self-regulation as one part of a complex set of governance [9,26,33-35], and consequently, for more dynamic and reflective approaches.

Various authors from diverse (northern and continental European) countries have highlighted important changes underway in healthcare that create new forms of professionalism in order to better fit contemporary healthcare needs, as discussed, for instance, by Plochg et al. [35], and also in relation to empirical findings mentioned in the previous section. Such new forms have been described variably as: ‘hybridisation’ [36], p. 761 or ‘organized professionalism…calling for multi-professional acts’ [37], p. 1360. ‘diversity of professionalism’ and flexibility between exclusionary and more ‘inclusive’ patterns [21], p. 221; ‘compatibility’ of different modes [28], p. 634 and ‘community professionalism’, as suggested by Tousijn [24], p. 533 with reference to Adler et al. [38]. Although the ‘labels’ differ, these approaches make much the same plea for overcoming the managerialism-professionalism dichotomy.

A static conception of the professionalism-managerialism relationship has also been questioned in organisational research [39-41]. Here, one important focus is on the ‘blurring of boundaries’ between professionalism, conceptualised as ‘internal’ mode of governing, and managerialism as an ‘external’ governance approach attempting to improve control and transparency of elitist professional knowledge. Waring and Currie [42], in their study of the management of knowledge around clinical risks in the NHS in the United Kingdom suggest ‘that doctors respond to change through a number of situated responses that limit managerial control over knowledge and reinforce claims to medical autonomy’ [42], p. 755. The authors use three categories to describe doctors’ responses: ‘co-optation’, ‘adaptation’ and ‘circumvention’. The findings reveal how ‘management techniques are co-opted into professional work as a form of resistance, with professionals being competent in management practice, rather than being co-opted into management roles’ [42], p. 774.

Cross-country comparative research has added strong evidence of the variety of changing professionalism-managerialism relationships. For example, Kirkpatrick and colleagues [16], in their comparison of medicine and management in England and Denmark, highlight the ways through which national institutions have shaped professional development and ‘that processes of re-stratification are more path dependent than is frequently acknowledged’ [6,16,33,34].

The merging processes between management and medicine have primarily been studied from the perspective of doctors and changing modes of professionalism, but the contemporary transformations do not travel on a ‘one-way road’ from management to medicine [12,15,43]. Von Knorring et al. [44], in their qualitative study of Swedish top managers, have revealed that top managers in healthcare tend to remake existing hierarchies between doctors and managers. In this study, the country council chief executive officers often perceived the management role in their organisations as weak. The authors could not identify a clear strategy of managing doctors but, instead, revealed four different strategies, and concluded that this pragmatic behaviour may in a longer perspective lead to a decrease in ‘the legitimacy of the manager role’ [44], p. 1. One important explanation for the persisting hierarchies might be the fact that managers cannot refer to a formalised knowledge system in the same way as doctors do [8,45].

In summary, the review of the literature reveals that ‘ownership’ of managerial tools is not naturally attached to management and managers, but can be used flexibly by different groups. Most importantly, there is no one uniform pattern of transformations and not ‘a’ new professionalism but various different ways of designing and re-designing professionalism and the relationship with organisations and management. This suggests a ‘need to overcome the hegemony/resistance framework in current analyses of the impact of management on professionalism’, as recently claimed by Numerato et al. [28], p. 626.

To put it more generally: dichotomous concepts are no longer sustainable. Instead, a more dynamic conceptual approach is needed that moves from the queries of ‘whether’ and ‘why’ the managerial-professionalism relationship is changing, towards the question ‘how’ this happens. This approach pays greater recognition to the contexts, the organisational settings and accountability structures, and consequently, creates new methodological challenges of researching diverse and ‘hybrid’ designs and comparing healthcare systems across organisational setting.

The problems of dichotomies and pre-existing categories in theorising new emergent relationships between medicine and management and their context-dependency direct us back to the need for empirical investigations. The major objectives to be addressed in this research are: to describe new emergent forms of control beyond the metaphor of hybridisation and to analyse context-specific conditions that may foster more integrated clinical management.

## Methods

Research was carried out in the European network on Medicine and Management (FP7 COST action ISA0903; http://www.dr-in-mgmt.eu) and includes Denmark, Germany, Greece, Netherlands, Poland, Portugal and Spain as country cases. As Burau [10], p. 569 highlights, more diverse comparisons have created ‘a range of challenges related to ensuring comparability, to comparing beyond the nation state, and to finding appropriate data for comparison’. Wrede [46] argues that case-based methods may help to capture complexity and, furthermore, highlights the benefits of interdisciplinary scholarship and methodological reflexivity. One example of a case study method is the so-called ‘decentred comparative research’ [47,48] that is characterised by qualitative methodology and interpretative analysis based on bottom-up developed categories and by high levels of sensitivity to the contexts. This approach seems to be useful also for the purpose of our research, because it minimises the constraints of research categories and theoretical bias, and therefore provides most opportunity for exploring emergent modes of control.

### The conceptual model

The research design is a case-based comparison comprising hospital case studies from seven European countries, namely Denmark, Germany, Greece, Netherlands, Poland, Portugal and Spain. These countries were selected for two reasons: at first and foremost, they represent a range of different health systems and economic and geopolitical conditions in Europe that made it possible to analyse both general trends and specific determinants of new modes of control. The cases also reflect existing expertise in the group of authors, and are therefore suitable for context-sensitive, qualitative case-based comparison.

**Figure 1:**
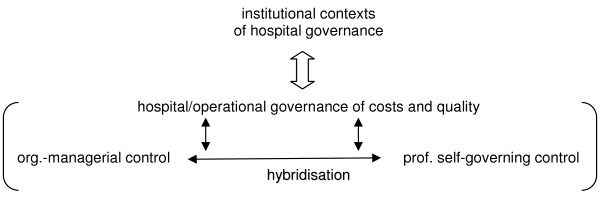
The conceptual model of researching control in hospitals.

The hospitals selected for inclusion here have broadly similar characteristics: they fall in the range of middle to large hospitals that are primarily public, have implemented a wide range of new managerial controls and, on average, achieved high standards of quality of the services provided when compared to other hospitals in the country. This selection of cases allows us to explore similarities and differences in the emergent patterns of control in relation to organisational settings without losing sight of national (macro-level) institutions. Figure 1 shows the conceptual model developed for our analysis.

We established an interdisciplinary research team comprising wide-ranging expertise from health management, economics and medicine to social and political science as well as from the different countries and from practice, research and policy. An interactive and reflective approach was applied both to the development of instruments and the analysis of data in comparative perspective: two workshops, each lasting a day and a half, and additional virtual discussion and comments from additional experts in the field were organised, providing the opportunity for an in-depth discussion and validation of indicators and findings. The major categories are summarised below and primarily include basic analytical categories drawn from the governance literature (as described previously) and in addition, an empirical category developed from our case study material.

*Governance*: serves as an umbrella concept including different forms (hierarchy, market, network, and professional self-governance), different sets of managerial and professional modes of governing, and institutions and actors:

● *Hierarchy*: top-down exercised on levels of state and/or hospitals.

● *Market:* all forms of competition; privatisation; consumer/patient choice.

● *Network*: complex forms of governing with higher levels of negotiations and more plural stakeholders involved, including among others, corporatist-style governance.

● *Professional self*-*governance*: the state has delegated regulatory powers and responsibility for public sector policy-making to professional associations.

*Accountability structures*: roughly described as ‘who does what, reporting to whom’.

*Coordination:* serves as an empirical category, including coordination across the levels of the hospital between top-down and bottom-up governing, and between managerial-organisational and professional self-governing procedures.

### Research instruments and data collection

A *Hospital Control Assessment Framework* (H-CAF) [49] was developed that served as guidance for the collection of data in our different countries. The H-CAF is a pioneering instrument, because no research exists that brings cross-country comparison to the level of the organisation. It is based on a semi-structured, extensive topic guide. Although the instrument is very detailed in order to assure comparability between countries and in an interdisciplinary group of authors, it is important to note that it served as a qualitative tool. Data were collected by the respective country experts in the group of authors using different sources of information. However, the assessment framework might serve as a pilot for developing a standardised questionnaire for surveying a larger number of hospitals in future.

Drawing on the conceptual model outlined in Figure 1, the H-CAF comprises the following five categories:

1. Key characteristics of the healthcare state and institutional contexts of hospital governance (macro-level);

2. Governance structures of the hospital (meso-level/organisation);

3. Financial/efficiency controls and managerial tools (organisational levels, accountability and actors);

4. Quality and safety controls and organisational-managerial tools (organisational levels; accountability and actors);

5. Professional/medical self-governing controls and tools (organisational levels; accountability and actors) [49].

A set of indicators was developed for each of the categories in order to specify the different tools of control in hospitals and how they are applied. While category 1 focuses on the macro-level of healthcare states and category 2 on the meso-level of organisations more generally, the remaining three categories seek to combine actor-centred and organisational dimensions, asking ‘who is responsible’ and at ‘what level of the organisation’. In relation to existing instruments [5,50], the innovative momentum of this instrument is a comparative analysis on the organisational level that, firstly, pays greater attention to professional self-governance and the connectedness between managerialism-professionalism, and, secondly, to actor-centred governance and accountability structures.

In every country included in our study, one hospital was selected as a case study following the criteria defined above. The case studies were collected between September and November 2011 and draw on document analysis and other secondary sources and expert information in accordance with ethical guidelines of the COST action and the respective national policies; ethics approval for expert information is not required in any of the countries where it has been included in this study.

A bottom-up approach and interpretative methodology were used to analyse the country cases. The findings were discussed in an interactive workshop, as already mentioned, firstly in relation to a single case study and then in comparative perspective. The analysis followed three main steps: (1) identifying common trends across the case studies, (2) describing the new emergent forms of control case by case and exploring similar patterns, and (3) finally developing an analytical category that helps to draw a comparative map of control across the levels and the substance of governance. This complex analytical procedure helps to improve both comparability and context-sensitivity. While the small-scale empirical basis (one case study per country) facilitates a qualitative analysis, it also has a number of limitations that are explained in more detail in the conclusion section.

## Results

### The country case studies

Table 1 provides an overview of the institutional contexts and the key characteristics of operational governance on the level of the hospitals. This is further explored in the case-by-case presentation by highlighting processes and actors involved in control.

**Table 1:** Hospital governance in European countries

**Countries**	**Institutional contexts**	**Hospital organisation**	**Financial controls**	**Managerial governance**	**Medical self-governance**
Denmark	decentralised, network-based governance embedded in hierarchy; little market & strong patient involvement	troika structure (medical, nursing, admin. directors) at top level indicative of all levels of management	mixed DRG system; centralised framework with some flexibility at all levels	strong bottom-up controls with integrated medical power on all levels; e.g. monitoring, quality reports, patient safety	important, but strongly integrated on all levels (see managerial governance)
Germany	decentralised corporatist-style governance; weak hierarchy & weak direct patient involvement; some market	troika structure at top level with some flexibility; little systematic implementation at department level & strong medical power	mixed DRG system, with some flexibility & strong involvement of doctor at all levels	mix of top-down & bottom-up controls with integrated medical power; high flexibility of doctors on department level	important on all levels, but strongest at department level; integrated with some flexibility of doctors, esp. quality & safety
Greece	hierarchy (some decentralisation) with market & corporatism; lack of patient involvement	appointed (Ministry) director with multi-prof. board, but lack of coordination with lower tiers; integration of doctors highly flexible	no DRG system; budget strongly hierarchical with little flexibility at department level; limited involvement of doctors	strongly top-down at the macro-level but limited between levels; new emergent quality controls primarily controlled by doctors	important and strongest at the department level; medical power separated & strong in the area of quality
Netherlands	mix of corporatism, hierarchy, market, with decentralisation & patient involvement; increasing market with strong insurers	partnership governance between administration & doctors with strong medical power on all levels	mixed DRG system; some diversity, increasingly moving towards performance-based cost controls	connected to benchmarks & public control with strong integrated medical power; increasingly demand-led	important in all areas; strongly integrated, e.g. education, new emergent specialty of medical management
Poland	centralised corporatism with some hierarchy & market; weak patient involvement	general director & co-directors (medicine, nursing, finance, logistics); some flexibility & diversity on the department level	DRG-based, some involvement of doctors but increasingly stronger hierarchy (centralised & hospital level)	top-down integration of doctors coexist with separation; connection to bottom-up controls is weak & highly diverse at department level	strong in clinical practice but weaker in cost controls; highly dependent on the level & subject; some voluntary efforts, e.g. guidelines
Portugal	hierarchy with corporatised public sector; some market, little patient involvement	troika structure at top level with some flexibility; little systematic implementation at department level; strong medical power	DRG system with strong involvement of doctors; some flexibility at all levels of the organisation	some top down controls but weak & poorly connected with bottom-up controls; lack of transparency; highly diverse	strong & decentralised; little compulsory performance management but voluntary efforts
Spain	strong (regional) hierarchy with incomplete integration of medical power; little patient involvement; increasing privatisation	troika structure relevant on all levels; but double structure of general & ‘doctors only’ boards assures flexibility & medical power	budget fixed by regional authority with some flexibility of hospitals; strong medical power at level of departments	top-down with some bottom-up controls; troika structure expanding, but quality mainly managed by doctors; weak coordination & flexibility	important, strongest in the area of quality & department level, e.g. CPD, guidelines; integration & separation are combined strategically

#### Denmark

Within the context of strong public responsibility for hospitals and strongly integrated and network-based governance arrangements on the level of regional authorities, a so-called ‘troika’ structure or ‘triumvirate’ is established at the level of the organisation, comprising a medical, nursing and administrative director. This structure is indicative for all levels of management. Strong bottom-up controls with highly integrated medical power and patient involvement serve as a blueprint for all new modes of control and regulatory bodies. This comprises both cost and quality management procedures, examples of which are a mixed DRG system, monitoring systems, quality reports, patient safety and complaints management. In this situation, new modes of control broaden both the inclusion of management into medicine (‘hybridisation’) and complex, plural modes of governance on all levels of the organisation. Organisational conditions are becoming increasingly relevant in defining the modes of control. Consequently, accountability structures are complex and coherent and, this in turn, limits the space for strategic action of doctors.

#### Germany

Decentralised, networked-based governance with strong medical power, some hierarchy and market and relatively weak (although increasing) patient involvement together with a mix of public, third sector and commercial hospitals provide the framework. A troika structure is established on the level of the hospital, while management arrangements at department levels show higher flexibility and diversity. The organisation has introduced a number of new managerial procedures that are more centralised for cost control (DRG system) in relation to quality and safety management with greater flexibility of departments. This has led to divergent transformations: top-down exercised governance may expand into medical domains on hospital level, while at the level of departments doctors may design clinical management filling in a regulatory gap that results from the absence of a comprehensive troika structure. New modes of performance management are established, but accountability structures are not adequately adapted. This creates space for strategic action of doctors as the most powerful group in hospital governance.

#### Greece

Hierarchy (with some decentralisation) and market together with separated medical power and lack of patient participation provide the institutional framework for hospital governance. Hospitals are primarily public and headed by a general director (appointed by the Ministry) and a multi-professional board that includes doctors. Budgets are fixed with little flexibility on the level of departments and no performance-based elements. The hospital shows a strong hierarchical management with some elements of corporatist governance and market, but an overall vacuum of both self-governing and managerial controls exists below the top level. In this situation, new forms of quality control are implemented top-down and primarily controlled by doctors, while medical integration is weak in the area of cost controls. The lack of performance management and comprehensive accountability structures combine to create highly diverse patterns of control. Consequently, there is an overall weak coordination between mixed elements of governance and strong medical power in the area of quality.

#### Netherlands

A complex and diverse mix of network, hierarchy and market with primarily public responsibility for hospitals, patient participation and strong elements of professional self-governance is relevant on all levels of governance. This has created a dynamic landscape of cost and quality management where professional self-regulatory powers are strongly integrated (e.g. mixed DRG system with increasingly relevant performance-based controls, medical checklists, inclusion of management in medical curricula) and, interestingly, a new specialty of medical management is emerging. Managerial procedures are increasingly demand-led, for instance, the hospital must set sound price and quality parameters to achieve efficient agreements with insurers, and doctors, in turn, must act within this framework and also respond to citizens’ demands. This makes the organisation a key arena of performance-based management procedures that are highly integrated in a coherent structure of accountability, thus reducing strategic action of a single actor, like doctors.

#### Poland

Centralised governance with strong corporatism, some market and weak patient involvement but primarily public responsibility for hospital services set the scene for control procedures. Centralised and organisational controls are flexibly combined; for example, some centralised (state level) rules apply for teaching hospitals and impact on staffing levels and the skill-mix. Medical self-regulatory procedures are important, but largely separated from management, although there are signs of emergent corporatist governance, like the establishment of a multi-professional board of directors or external expert reports. Hence, this structure currently lacks of coherence at the level of departments. In this situation, centralised managerial controls increasingly intervene in medical budgets, thus reducing medical power, while organisational controls are weaker and flexibility of doctors higher in the area of quality and safety management. Consequently, accountability structures are highly diverse and this creates uncertainty and variety of medicine and management relationships.

#### Portugal

Hospital governance is characterised by hierarchy with strong medical power across all levels of governance and a corporatised public sector, increasing marketisation but lacking citizen/patient involvement and more complex forms of partnership governance. There are some signs of more centralised control on the top level of the hospital, composed of a troika structure, like the appointment of the hospital director and the establishment of a DRG system. Yet these attempts remain largely disconnected from the lower levels of governance, where control is poorly developed. Consequently, top-down introduced managerial controls may meet with a strong system of professional self-governance at the level of departments. On the backdrop of weak state and patient control of hospitals, the organisation is gaining significance. In a situation of poorly established accountability structures, the ability of doctors to use management strategically is increasing on the level of departments and in the area of quality management.

#### Spain

Strong decentralised (regional) hierarchy with structurally integrated medical power, public responsibility for hospitals with some privatisation and overall weak patient involvement provide the framework for hospital governance. The hospital has established a troika structure that is relevant on all levels, but the general boards coexist with ‘doctors only’ boards and create a double structure of highly integrated, and at the same time, separated medical power. Fixed budgets have been introduced top-down with some flexibility on department levels. A wide range of new controls, including incentivised Continuing Professional Development (CPD) and performance measurements, have been established and connected through macro-meso-micro level contractualisation. Medicine is expanding into management and this is driven by both departmentalisation and the establishment of clinical management with doctors utilising control bottom-up. The double structure of integration and separation allows for high flexibility and strategic action of doctors, but new emergent accountability structures may limit these options.

## Discussion

### Mapping the emergent forms of control in comparative perspective

Across our case studies, a general trend towards mixed forms of governing and more integrated modes of managerial and professional self-governing controls are confirmed and add further evidence to the scholarly debate over changing hospital governance [1]. The empirical findings support our theoretical argument for an end to dichotomous concepts of contradicting logics of management and medicine/professionalism, highlighting that ‘a manager is sitting in the minds’ – as we put it in our metaphor – rather than exercising external control of doctors. However, our case studies reveal high variations in the ways control is exercised on the level of the hospital and this points to the relevance of organisational settings. Three different patterns of emergent controls can be identified.

Pattern 1 ‘integrated control’: characterised by strong integration, and complex and coherent coordination between medicine and management, as found in Denmark and the Netherlands. Complex accountability structures limit strategic action of doctors as well as ad-hoc interest-driven hierarchical interventions of governments and organisations. Here, transformations occur across the areas of costs and quality-safety control, thus creating new forms of control in-between ‘management-medicine’, as, for instance, the attempt to establish a new speciality of medical management in the Netherlands illustrates.

Pattern 2 ‘partly integrated control’: medical power is strongly integrated in the modes of control, but coordination between control on the levels of the hospital and the departments shows gaps and variations. This pattern of control may show up as ‘double structure’ of integrated and separated medical power, like in Spain, or as limited/weak troika structure on the level of departments in Germany. In both cases, medical power is constrained in the area of cost controls and on the top level of hospital management, while quality and safety controls provide higher flexibility and opportunities for strategic action of doctors. More integrated patterns of medicine and management are emerging, but transformations are diverse and uneven.

Pattern 3 ‘fragmented control’: integration of medicine and management is limited and coordination overall weak, as observed in Portugal, Poland and Greece. Although there is some variation among these countries, all show efforts towards improved coordination and integration. Characteristically, accountability structures are diverse, but variation and flexibility are highest at department level. This pattern provides most opportunity for strategic action of a single actor. In this situation doctors are most powerful in the area of quality and increasingly integrate new managerial tools, while developments on the top level and in the area of cost control limit medical power and may instead support state or hospital actors. There are some signs of integration of management in medicine in the area of quality, but a wide gap exists between the costs and quality controls. Transformations are generally highly disparate and uneven. This, in turn, may create uncertain and inefficient modes of control in hospitals.

Finally, our mapping exercise brings the relevance of coordination into perspective that makes it possible to empirically define and specify the level of ‘integration’ between management and professionalism in vertical (macro-level/state and hospital governance; top-level and department level of the organisation) and horizontal (costs and quality) directions.

#### Methodological considerations

There are also important limitations, since the study is explorative in nature and the empirical material is based on the assessment of one hospital per country. First, we have selected one type of hospital in European countries and used a case study design to develop reliable instruments and keep the dimensions of meso-level comparison manageable. While this type of public hospital is important in all countries included in our study, entrepreneurial forms, privatisation and third sector involvement are gaining ground and this creates an increasingly diverse landscape of hospitals [1,27,41]. Second, we focused on doctors and medical self-governance, but tasks and the composition of skills are increasingly re-designed and nurses, especially, are making inroads in clinical management [51-53].

## Conclusions

This article has set out to map new emergent forms of control in European hospitals in comparative perspective, with a particular focus on doctors and the changing relationship between medicine and management that are often described as hybridisation. We have argued the need for theoretical and methodological revisions that are better able to respond to contemporary trends towards mixed forms of hospital governance and a flexible use of managerial-organisational and professional self-governing controls [50,53]. Against this backdrop we have introduced a conceptual model building on a continuum of managerialism-professionalism controls, and have developed an assessment framework [49] that served to map new emergent forms of control in the context of organisational settings, thereby expanding cross-country comparison beyond governmental bodies.

One key conclusion drawn from our study in European hospitals is that managerial control is not simply an external force but, increasingly, is internalised ‘in the minds of doctors’. This finding is also supported by recent statements from representatives of hospital doctors that highlight management as a new chance for doctors [54,55].

Other conclusions are that the changing relationships of medicine and management play out differently and that organisations increasingly matter in setting the scene for new emergent controls [56]. Here, our research is able to contribute new knowledge to an emergent debate into medical leadership and governance innovation [3,16,57-60] and to put ‘flesh to the bones’ of what is commonly described as ‘hybridisation’ of medicine and management. Three patterns are emerging:

● ‘Integrated’ control with coherent coordination within the hospital and similar patterns for cost and quality controls;

● ‘Partly integrated’ control with diversity of coordination on hospital and department level and some fragmentation of cost and quality controls; and

● ‘Fragmented’ control with uneven and limited coordination within the hospital and a gap between quality more strongly controlled by medicine, and cost control by management.

The comparative approach and our empirical material here highlight the crucial relevance of ‘coordination’ of medicine and management across the levels (hospital/department) and the substance (cost/quality-safety) of governance. As an empirical category, ‘coordination’ is a strong indicator of new modes of control that may serve as taxonomy for future research in this area. Importantly, this indicator is robust and reliable across healthcare systems in Europe. We therefore suggest a new taxonomy of clinical management building on the question, ‘how coordinated are the new models of control in relation to the levels and the substance of coordination?’

Our empirical findings challenge contemporary healthcare policies that are usually concerned with instruments and outcome measurements and that too often rely on single interventions and the management of costs. Here, the research may highlight new avenues for hospital governance that put greater attention towards structural improvement of organisational settings that foster coherent modes of coordination of medicine and management.

## Competing interests

The authors declare that they have no competing interest.

## Authors’ contributions

EK has coordinated the study, developed a theoretical approach and drafted and revised the paper. All authors have carried out a country case study, contributed to the development of the research instrument (H-CAF) and the analysis in comparative perspective, and read and approved the final manuscript.

## Pre-publication history

The pre-publication history for this paper can be accessed here:

http://www.biomedcentral.com/1472-6963/13/246/prepub
